# Clinical Outcomes and Prognostic Factors in Uterine Adenosarcoma: A Multicenter Retrospective Cohort Study

**DOI:** 10.3390/cancers18182903

**Published:** 2026-09-08

**Authors:** Atacem Mert Aytekin, Ipek Betul Ozcivit Erkan, Seyma Okumus, Bilgehan Saglik, Ayse Yavuz, Utku Akgor, Onur Can Zaim, Mert Urfalioglu, Banu Boso Aslantas, Hamdullah Sozen, Ghanim Khatib, Ilkbal Temel Yuksel, Merve Aldikactioglu Talmac, Abdullah Serdar Acikgoz, Tugan Bese, Oguzhan Kuru

**Affiliations:** 1 Division of Gynecologic Oncology, Department of Obstetrics and Gynecology, Cerrahpasa Faculty of Medicine, Istanbul University-Cerrahpasa, 34098 Istanbul, Türkiye; bilgeesaglik@gmail.com (B.S.); aysyvvz@gmail.com (A.Y.); abdullahserdar.acikgoz@iuc.edu.tr (A.S.A.); tuganbese@gmail.com (T.B.); droguzhankuru@yahoo.com (O.K.); 2 Department of Obstetrics and Gynecology, Cerrahpasa Faculty of Medicine, Istanbul University-Cerrahpasa, 34098 Istanbul, Türkiye; ipekbetulozcivit@gmail.com (I.B.O.E.); okumusseymaa@gmail.com (S.O.); 3 Department of Obstetrics and Gynecology, Health Sciences University, Istanbul Training and Research Hospital, 34098 Istanbul, Türkiye; 4 Division of Gynecologic Oncology, Department of Obstetrics and Gynecology, Hacettepe University, 06230 Ankara, Türkiye; utkuakgor@gmail.com (U.A.); oczaim@yahoo.com.tr (O.C.Z.); merturfalioglu@gmail.com (M.U.); 5 Division of Gynecologic Oncology, Department of Obstetrics and Gynecology, Baskent University Adana Hospital, 01140 Adana, Türkiye; banuboso@hotmail.com; 6 Division of Gynecologic Oncology, Department of Obstetrics and Gynecology, Istanbul Faculty of Medicine, Istanbul University, 34093 Istanbul, Türkiye; drhsozen@gmail.com; 7 Division of Gynecologic Oncology, Department of Obstetrics and Gynecology, Cukurova University Faculty of Medicine, 01330 Adana, Türkiye; ghanim.khatib@gmail.com; 8 Division of Gynecologic Oncology, Department of Obstetrics and Gynecology, Basaksehir Cam and Sakura City Hospital, 34480 Istanbul, Turkey; drilkbaltemel@gmail.com; 9 Division of Gynecologic Oncology, Department of Obstetrics and Gynecology, Health Sciences University, Istanbul Kanuni Sultan Suleyman Training and Research Hospital, 34303 Istanbul, Turkey; drmrve@hotmail.com

**Keywords:** uterine adenosarcoma, sarcomatous overgrowth, lymphovascular space invasion, prognosis, disease-free survival, overall survival

## Abstract

**Background/Objectives:** Uterine adenosarcoma is a rare uterine malignancy with limited evidence on prognostic factors and long-term outcomes. We aimed to evaluate clinicopathological characteristics, treatment patterns, oncologic outcomes, and prognostic factors associated with disease-free survival (DFS) and overall survival (OS) in patients with uterine adenosarcoma. **Methods:** This multicenter retrospective cohort study included 43 patients with histopathologically confirmed uterine adenosarcoma who underwent primary surgery at seven tertiary referral centers in Türkiye between 2016 and 2026. Clinicopathological features, treatment modalities, recurrence patterns, and survival outcomes were assessed. Survival was analyzed using Kaplan–Meier and log-rank methods, and multivariable Cox proportional hazards regression was performed to identify independent prognostic factors. **Results:** The mean age at diagnosis was 59.1 ± 10.8 years, and 83.7% of patients were postmenopausal. Pelvic pain (62.8%) and abnormal uterine bleeding (60.5%) were the most common presenting symptoms. Most patients (79.1%) underwent laparotomy, and 79.1% had stage I disease. Sarcomatous overgrowth (SO) was present in 44.1% and lymphovascular space invasion (LVSI) in 18.6%. During a median follow-up of 72 months, 34.9% experienced recurrence, most commonly in the pelvis. The five-year DFS and OS rates were 59.9% and 80.4%, respectively. SO was independently associated with DFS (HR 4.41, 95% CI 1.21–16.08; *p* = 0.025) and OS (HR 10.23, 95% CI 1.16–89.80; *p* = 0.036), while LVSI was independently associated with OS (HR 11.17, 95% CI 1.34–93.14; *p* = 0.026). **Conclusions:** Uterine adenosarcoma showed favorable long-term survival but substantial recurrence risk. SO and LVSI may have potential prognostic relevance and could contribute to postoperative risk assessment and individualized follow-up.

## Simple Summary

Uterine adenosarcoma is a rare type of cancer of the uterus. This study looked at the clinical features, treatments, recurrence, and long-term outcomes of 43 women diagnosed with this cancer in seven hospitals in Türkiye between 2016 and 2026. Most women were postmenopausal and had early-stage disease at diagnosis. The most common symptoms were pelvic pain and abnormal uterine bleeding. During an average follow-up of 6 years, about one-third of the women developed a recurrence, most often in the pelvis. Overall, long-term survival was favorable, with about 80% of women remaining alive 5 years after diagnosis. However, certain tumor features were associated with a higher risk of recurrence and death. In particular, sarcomatous overgrowth and lymphovascular space invasion were linked to poorer outcomes. These findings suggest that these tumor features may help doctors identify women who require closer follow-up and more individualized postoperative care.

## 1. Introduction

Uterine sarcomas comprise a diverse group of mesenchymal malignancies arising from the myometrium or endometrial stroma and account for <10% of all uterine malignancies [1,2]. Uterine adenosarcoma is an uncommon biphasic tumor characterized by a benign epithelial component and a malignant stromal component. It accounts for approximately 5–9% of uterine sarcomas and 0.2% of all uterine malignancies [3,4]. Adenosarcoma is primarily diagnosed based on characteristic morphological features, as no specific immunohistochemical marker has been identified [3,5]. Although adenosarcoma most commonly arises in the uterus, rare cases involving the ovary, cervix, and other sites have also been reported [6,7,8,9].

Uterine adenosarcomas generally has a favorable prognosis, with most patients diagnosed at an early stage [10,11]. However, clinical outcomes are heterogeneous, and some tumors exhibit aggressive behavior. Sarcomatous overgrowth (SO), defined as a sarcomatous component exceeding 25% of the tumor, is one of the most pathological features associated with aggressive biological behavior and poor clinical outcomes [12,13,14,15,16]. Although several clinicopathological factors, including International Federation of Gynecology and Obstetrics (FIGO) stage, myometrial invasion, lymphovascular space invasion (LVSI), adnexal involvement, and extrauterine disease, have been associated with clinical outcomes, their prognostic significance has varied across retrospective studies, reflecting the rarity and heterogeneity of this tumor [3,17]. Identifying prognostic factors in uterine adenosarcoma is therefore essential for accurate risk stratification and the development of individualized treatment strategies.

Surgical management, typically involving hysterectomy with bilateral salpingo-oophorectomy, is the primary treatment approach. However, the therapeutic value of lymphadenectomy and adjuvant therapy remains uncertain [18,19,20]. Current evidence is largely derived from retrospective studies with limited sample sizes and heterogeneous management strategies, highlighting the need for additional multicenter data to improve prognostic assessment and guide individualized management [11,19,21].

Therefore, we aimed to evaluate clinicopathological characteristics, treatment approaches, and long-term oncologic outcomes, including disease-free survival (DFS) and overall survival (OS), in a multicenter cohort of patients with uterine adenosarcoma treated at seven tertiary referral centers in Türkiye. We further investigated clinical and histopathological factors associated with survival outcomes to identify the potential prognostic markers.

## 2. Materials and Methods

### 2.1. Study Design and Setting

This study followed the STROBE recommendations [22]. Ethical approval was obtained from the Institutional Ethics Committee (Approval Date: 14 May 2026; Approval No.: 1636102). Patients with histopathologically confirmed uterine adenosarcoma who underwent surgical treatment between January 2016 and May 2026 at seven tertiary referral gynecologic oncology centers (Istanbul University-Cerrahpasa, Hacettepe University, Baskent University Adana Hospital, Istanbul Faculty of Medicine, Cukurova University, Basaksehir Cam and Sakura City Hospital and Istanbul Kanuni Sultan Suleyman Training and Research Hospital) in Türkiye were eligible for inclusion. The analysis included all patients who fulfilled the prespecified eligibility criteria within the study period.

Because of the retrospective multicenter design, there was no uniform protocol for surgical management, pathological assessment, adjuvant treatment, or follow-up across participating centers during the study period. Management decisions were made according to institutional practice and individual patient and tumor characteristics.

### 2.2. Participants and Data Collection

Patients were identified through institutional electronic medical records and pathology databases. Patients were excluded if histopathological confirmation of uterine adenosarcoma was unavailable, primary surgical treatment was not performed at one of the participating centers, follow-up data were insufficient for survival analysis, or essential clinicopathological information was unavailable. Demographic, clinical, pathological, treatment, follow-up, and survival data were retrospectively extracted from patient medical records using a predefined data collection template. Histopathological diagnoses were based on the original pathology reports, and relevant pathological characteristics were recorded from the available surgical specimens.

### 2.3. Variables

The collected variables were as follows: age, body mass index (BMI), gravidity, parity, menopausal status, presence of concomitant gynecologic pathology, surgical approach, type of surgery, tumor size, pregnancy status, comorbidities, preoperative sampling method, date of surgery, presenting clinical findings (abnormal uterine bleeding, pelvic pain, and a history of endometrial polyp), histopathological characteristics including LVSI, myometrial invasion (MI), serosal involvement, endocervical involvement, SO, adnexal involvement, lymph node metastasis, residual disease, FIGO stage, adjuvant treatment modality and regimen, total follow-up duration, recurrence status, time to recurrence, treatment for recurrent disease, and survival status with date of death. SO was defined as a sarcomatous component comprising more than 25% of the tumor. LVSI was defined as the presence of tumor cells within lymphatic and/or vascular spaces. Histopathological diagnoses and pathological characteristics were obtained from the original pathology reports and available surgical specimens. No centralized pathological review was performed across participating centers. Tumors were staged according to the 2009 FIGO staging system for uterine adenosarcoma [23]. Recurrence was defined as radiologically and/or histopathologically documented evidence of disease following completion of primary treatment. The primary oncologic outcomes were DFS and OS. Disease-free survival (DFS) was calculated from completion of the initial treatment until the first documented recurrence or the last available follow-up [24]. Overall survival (OS) was measured from the time of diagnosis to death from any cause or the most recent follow-up, whichever occurred first [25].

### 2.4. Sample Size Calculation and Statistical Analysis

Given the retrospective nature of the study, an a priori sample size calculation was not undertaken. Instead, all patients who satisfied the predefined eligibility criteria during the study period were included in the analysis.

The distribution of continuous variables was evaluated using the Shapiro–Wilk test. Normally distributed data were summarized as mean ± standard deviation (SD), while non-normally distributed data were reported as median values with the corresponding minimum and maximum. Categorical variables were summarized as counts and percentages.

Survival analyses were estimated via the Kaplan–Meier method, and survival curves were evaluated via the log-rank test. Mean survival estimates were presented with their standard errors (SEs). Associations between clinicopathological characteristics and DFS or OS were first examined in univariable analyses. Variables demonstrating significant associations in the initial analyses were subsequently evaluated in multivariable Cox regression models. FIGO stage was not included in the Cox regression analysis because it is associated with adnexal involvement and lymph node status. The findings are reported as hazard ratios (HRs) with corresponding 95% confidence intervals (CIs).

For missing observations, analyses were conducted using the available data, and the number of participants contributing to each analysis was reported when relevant. Participants with no recorded recurrence were censored at their most recent follow-up assessment.

Statistical analyses were conducted with IBM SPSS Statistics for Windows and MEDCALC v23.7.1, version 25.0 (IBM Corp., Armonk, NY, USA). Statistical significance was defined as a two-tailed *p* value below 0.05.

## 3. Results

A total of 43 patients from seven tertiary referral centers were included in the analysis. The mean age at diagnosis was 59.09 ± 10.75 years, and the median BMI was 30.80 kg/m^2^ (range, 20.57–52.80) (Table 1). Most patients (83.7%) were postmenopausal. The most common presenting complaints were pelvic pain (62.8%, *n* = 27) and abnormal uterine bleeding (60.5%, *n* = 26). A history of endometrial polyp was present in 34.9% of patients. Preoperative endometrial sampling was performed in 83.7% of patients, most commonly dilatation and curettage (D&C) (52.8%), or Pipelle biopsy (30.6%) (Table 1).

Most patients underwent laparotomy (79.1%), whereas 20.9% underwent minimally invasive surgery (only one of them underwent robot-assisted surgery) (Table 2). 21 patients (48.8%) underwent hysterectomy with or without bilateral salpingo-oophorectomy (BSO), while seven (16.3%) also had combined pelvic ± para-aortic lymphadenectomy. 11 of them (25.6%) underwent hysterectomy with or without BSO combined with pelvic ± para-aortic lymphadenectomy and omentectomy, and four patients (9.3%) underwent cytoreductive (debulking) surgery. Nine patients (20.9%) underwent pelvic lymphadenectomy alone, whereas 13 patients (30.2%) underwent both pelvic and para-aortic lymphadenectomy (Table 2).

Thirty-four patients were classified as stage I, four as stage II, and five as stage III (Table 3). Peritoneal cytology was available for 16 patients, and all results were reported no malignancy. Deep myometrial invasion, serosal involvement, endocervical involvement, LVSI, adnexal involvement and lymph node metastasis were observed in 30.2%, 14.0%, 16.3%, 18.6%, 9.3%, and 9.3% of patients, respectively. The most common concomitant gynecologic pathology was leiomyoma (41.9%), followed by adenomyosis (25.6%). No concomitant gynecologic pathology was identified in 30.2% of patients.

The immunohistochemical profiles of the tumor components were evaluated in cases with available data (Supplementary Table S1). The median mitotic index was 7/10 high-power fields (HPFs) (range, 1–35). Estrogen receptor (ER) expression and progesterone receptor (PR) expression were evaluated separately in the epithelial and sarcomatous components, with median ER/PR expression of 80%/60% and 40%/15%, respectively. Ki-67 proliferation index was 23.63% ± 17.50% in the epithelial component and 20% (range, 1–80%) in the sarcomatous component. CD10 expression was positive in 26/29 cases (89.7%). Cyclin D1 expression was assessed in seven epithelial and 11 sarcomatous components, with positivity rates of 42.9% and 27.3%, respectively. Vimentin expression was evaluated in three epithelial and four sarcomatous components and was positive in two (66.7%) and three (75.0%). cases, respectively.

Of the 43 patients included in the analysis, 24 (55.8%) underwent adjuvant treatment following surgery. This comprised chemotherapy alone in 32.6%, radiotherapy alone in 16.3%, and combined chemoradiotherapy in 7.0% of patients. Doxorubicin was the most common administered regimen (*n* = 10, 58.8%) (Table 4). During follow-up, disease recurrence occurred in 15 patients (34.9%), most commonly in the pelvic (*n* = 9, 60.0%), followed by the thorax (*n* = 3, 20.0%). Treatment for recurrent disease included secondary cytoreductive surgery (46.2%, *n* = 6), chemotherapy (38.5%, *n* = 5), and radiotherapy (15.4%, *n* = 2). Two patients with recurrent disease died before receiving any treatment because of poor performance status and rapidly progressive disease. Following treatment for the first recurrence, one patient (4.3%) developed a second recurrence. During the follow-up period, 10 patients died (23.3%).

### 3.1. Oncologic Outcomes and Survival Analysis

The median follow-up period after surgery was 72 months (SE = 11.36). The mean DFS for the entire cohort was 111.93 ± 12.02 months (95% CI, 88.37–135.49). Kaplan–Meier analysis demonstrated 1-, 3-, and 5-year DFS rates of 83.0%, 69.9%, and 59.9%, respectively. The mean OS was 159.56 ± 13.88 months (95% CI, 132.36–186.76). The corresponding 1-, 3-, and 5-year OS rates were 92.9%, 80.4%, and 80.4%, respectively.

#### 3.1.1. Univariate Analysis of Prognostic Factors Associated with Survival

**Figure 1:**
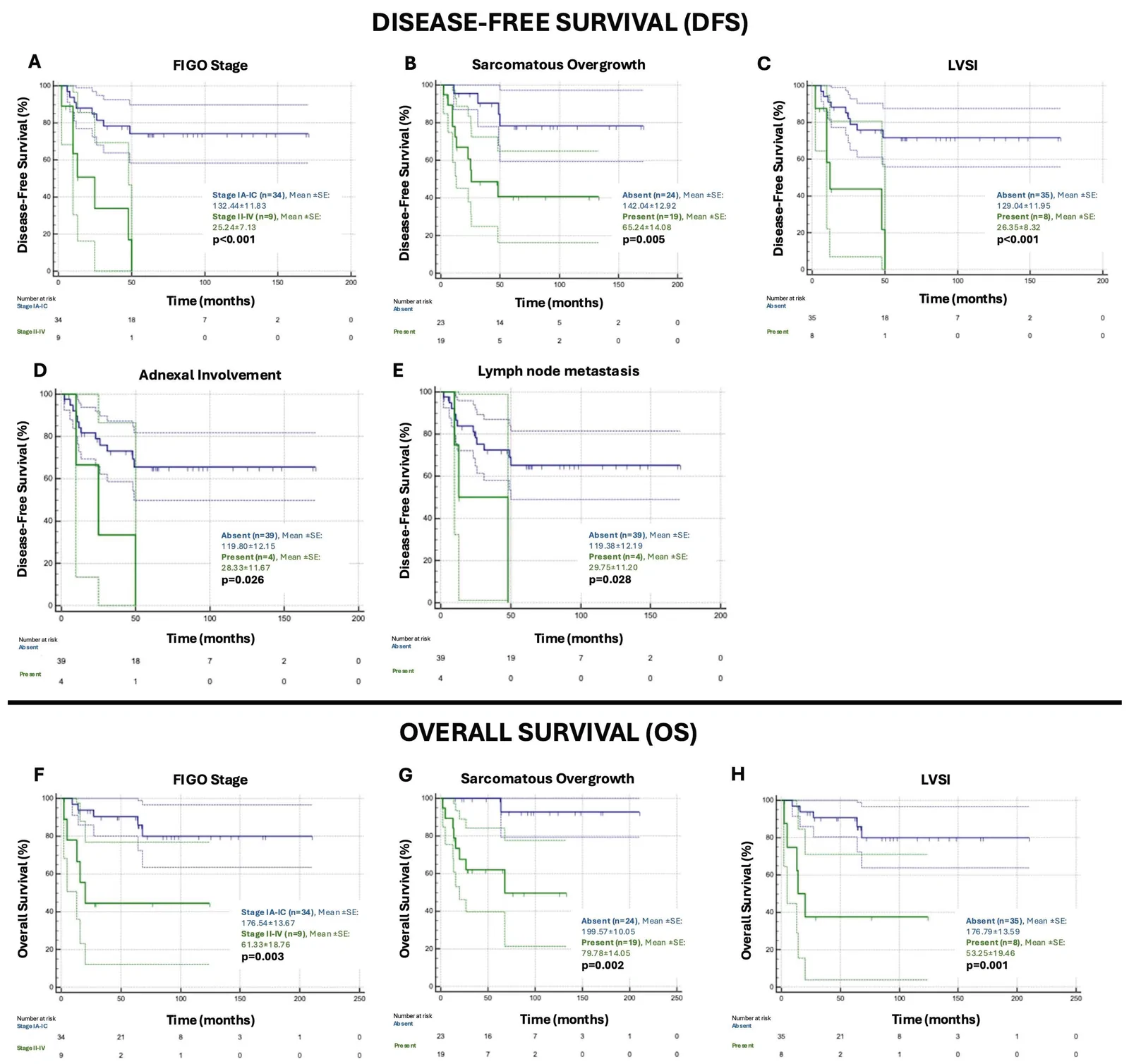
Kaplan–Meier survival curves for disease-free survival (DFS) and overall survival (OS) according to clinicopathological prognostic factors in patients with uterine adenosarcoma. Disease-free survival (DFS) curves stratified by (**A**) FIGO stage, (**B**) sarcomatous overgrowth, (**C**) lymphovascular space invasion (LVSI), (**D**) adnexal involvement, and (**E**) lymph node metastasis. Overall survival (OS) curves stratified by (**F**) FIGO stage, (**G**) sarcomatous overgrowth, and (**H**) lymphovascular space invasion (LVSI). SE: standard error.

Univariate survival analysis using the log-rank test demonstrated that FIGO stage (stage IA–IC vs. higher stages; *p* < 0.001), the presence of SO (*p* = 0.005), LVSI (*p* < 0.001), adnexal involvement (*p* = 0.026), and lymph node metastasis (*p* = 0.028) were significantly linked to DFS. The mean DFS was 132.44 ± 11.83 months in patients with early-stage disease (FIGO IA–IC), compared with 25.24 ± 7.13 months in those with advanced-stage disease. Similarly, patients without SO had a mean DFS of 142.04 ± 12.92 months, whereas those with SO had a substantially shorter mean DFS of 65.24 ± 14.08 months (Figure 1).

In the analysis of OS, FIGO stage (*p* = 0.003), the presence of SO (*p* = 0.002), and LVSI (*p* = 0.001) were identified as significant prognostic factors. In contrast, depth of myometrial invasion, tumor size, and the presence of tumor necrosis were not significantly associated with either DFS or OS (Table 5).

#### 3.1.2. Multivariate Cox Regression Analysis of Prognostic Factors Affecting Survival

Clinicopathological variables demonstrating significance in the univariable analysis were subsequently evaluated in a multivariable Cox regression model for DFS. Among the factors examined, SO was the only variable that retained independent prognostic significance (HR 4.41, 95% CI 1.21–16.08; *p* = 0.025), corresponding to an approximately 4.4-fold higher hazard of recurrence. No independent association with DFS was observed for LVSI, adnexal involvement, or lymph node metastasis (Table 5).

In the multivariate Cox regression model for OS, both SO (HR = 10.23, 95% CI: 1.16–89.80, *p* = 0.036) and LVSI (HR = 11.17, 95% CI: 1.34–93.14, *p* = 0.026) remained statistically associated with OS. However, these estimated should be interpreted cautiously given the small number of events and wide CIs. In contrast, adnexal involvement and lymph node metastasis did not show an independent association with OS.

## 4. Discussion

In this retrospective multicenter cohort analysis of 43 patients with uterine adenosarcoma from seven tertiary referral centers across Türkiye, we evaluated clinicopathological characteristics, treatment patterns, and long-term oncologic outcome. To our knowledge, this represents the largest multicenter uterine adenosarcoma series reported from Türkiye and one of the largest multicenter cohorts reported internationally. Although most patients had early-stage disease; adverse pathological features, including deep myometrial invasion, SO, LVSI, and lymph node involvement, were observed in a substantial proportion of cases. Over a median follow-up period of 72 months, recurrence occurred in approximately one-third of patients, with the pelvis being the most frequent site of relapse. The 5-year DFS and OS rates were 59.9% and 80.4%, respectively. Importantly, SO was independently associated with both DFS and OS, while LVSI was independently associated with OS. These findings suggest that SO may have prognostic relevance in uterine adenosarcoma and may be considered as a potential factor in risk stratification and prognostic assessment.

Our study showed that patients with early-stage disease (FIGO IA–IC) demonstrated significantly better DFS and OS than those with advanced-stage disease. This finding is consistent with previous studies identifying disease stage as one of the most important determinants of prognosis in uterine adenosarcoma. Arend et al. and Carroll et al. similarly reported favorable long-term survival in patients presenting with early-stage disease, whereas advanced-stage disease was associated with substantially higher risks of recurrence and mortality [10,15]. Smaller case series have reported similar findings [17,26]. Furthermore, the current guidelines also emphasized that most uterine adenosarcomas are diagnosed at an early stage and that surgical management generally provides favorable outcomes [13]. Consistent with these observations, the mean DFS exceeded 132 months among patients with early-stage disease in our cohort.

SO showed the strongest association with survival outcomes in our cohort and remained statistically associated with both DFS and OS in the multivariable models. In the multivariable models, SO was associated with a higher hazard of recurrence (HR 4.41, 95% CI 1.21–16.08) and death (HR 10.23, 95% CI 1.16–89.80). However, these estimates should be interpreted cautiously given the limited number of events and the wide Cis, which indicate substantial uncertainty and limited precision of these estimates, particularly for OS. Accordingly, these findings should be considered exploratory. Nevertheless, these results agree with earlier reports indicating that SO is linked to more aggressive tumor behavior, increased recurrence, shorter DFS, and reduced OS [10,12,15,16,27]. The association of SO with increased stromal proliferation, higher mitotic activity, and invasive behavior may explain its adverse prognostic significance. Thus, SO may have potential prognostic relevance for postoperative risk assessment and individualized follow-up, although this association requires confirmation in larger, independent cohorts.

In this cohort, LVSI was associated with an increased hazard of death in the multivariable model (HR 11.17, 95% CI 1.34–93.14); however, the wide CI and small number of deaths warrant cautious interpretation. Its lack of independent association with DFS may reflect the limited sample size and the relatively small number of recurrence events, as well as its frequent coexistence with other aggressive histopathological features, particularly SO. Nevertheless, our findings further support the prognostic relevance of LVSI, particularly for OS consistent with previous literature [15,28].

Other pathological factors, including depth of myometrial invasion, tumor size, and tumor necrosis were not independently associated with DFS or OS. Although adnexal involvement and lymph node metastasis were significantly associated with DFS in the univariate analysis, these associations were not retained after multivariate adjustment. Previous studies have reported conflicting findings regarding the prognostic significance of these factors [15,17,29]. Their independent effects may be difficult to distinguish from advanced stage and SO.

The 5-year DFS and OS rates were 59.9% and 80.4% observed in our cohort indicate that, despite a substantial risk of recurrence, long-term OS may remain favorable. Primary surgical management is centered on total hysterectomy, while the potential benefit of postoperative adjuvant treatment remains unclear, because of the absence of high-level evidence. Consistent with current ESGO/EURACAN/GCIG and UK guidelines, routine adjuvant therapy is not recommended, and treatment should be individualized through multidisciplinary assessment [11,30].

Among the strengths of this study are its multicenter design and the relatively substantial sample size given the rarity of this malignancy, long-term follow-up, and evaluation of prognostic factors using multivariate survival analyses. However, several limitations should be considered when interpreting our findings. The retrospective multicenter design may have introduced selection bias, as the participating institutions were tertiary referral centers and may have had differences in patient referral patterns and disease characteristics. Information bias is also possible because clinical, pathological, treatment, and follow-up data were retrospectively obtained from existing medical records, resulting in missing or inconsistently documented variables. Furthermore, heterogeneity in surgical management and adjuvant treatment approaches across participating centers may have influenced oncologic outcomes and limited the comparability of treatment strategies. The limited sample size, the predominance of early-stage disease in our cohort and these sources of heterogeneity may have reduced statistical power and limited the generalizability of our findings. In addition, immunohistochemical analyses were available only for a subset of patients, limiting the interpretation of biomarker findings. Furthermore, no centralized pathological review was performed, and pathological assessments were based on the original reports from participating institutions; therefore, interobserver variability in the assessment of SO, LVSI, and other histopathological features cannot be excluded.

Another important limitation of the present study is the small number of outcome events, particularly deaths, relative to the number of covariates evaluated in the multivariable Cox regression models. This creates a potential risk of model overfitting and may result in unstable estimates, as reflected by the wide CIs of some HRs. Therefore, the multivariable findings, particularly those for OS, should be interpreted as exploratory rather than definitive. The observed associations of SO and LVSI with survival require confirmation in larger, preferably prospective or larger collaborative multicenter cohorts with a greater number of outcome events. Despite these limitations, our study provides additional multicenter evidence regarding the prognostic determinants and long-term oncologic outcomes in uterine adenosarcoma.

## 5. Conclusions

In conclusion, uterine adenosarcoma is a rare gynecologic malignancy with relatively favorable OS but a substantial risk of recurrence. Within our study population, SO demonstrated the strongest independent association with both DFS and OS, while LVSI remained independently associated with OS. These findings suggest that SO and LVSI may have prognostic relevance and could contribute to postoperative risk assessment; however, given the small number of outcome events and the uncertainty of the estimates, these associations should be interpreted cautiously and require validation in larger multicenter cohorts.

## Figures and Tables

**Table 1:** Baseline demographic, clinical, and preoperative characteristics of patients with uterine adenosarcoma (N = 43).

Variables			Mean ± SD or Median (Min–Max) or *n* (%)
Age (years)			59.09 ± 10.75
Gravidity/Parity			4 (0–15)/3 (0–12)
BMI (kg/m^2^)			30.80 (20.57–52.80)
Menopausal Status	Premenopausal		7 (16.3)
	Postmenopausal		36 (83.7)
Clinical Presentation	Abnormal Uterine Bleeding (AUB)	Absent	17 (39.5)
		Present	26 (60.5)
	Pelvic Pain	Absent	16 (37.2)
		Present	27 (62.8)
	Previous History of Endometrial Polyp	Absent	28 (65.1)
		Present	15 (34.9)
Preoperative Biopsy Sampling	Not Performed		7 (16.3)
	Pipelle endometrial biopsy		11 (30.6)
	Dilatation and Curettage (D&C)		19 (52.8)
	Hysteroscopy		6 (16.7)

BMI: body mass index, Min: minimum, Max: maximum, SD: Standard Deviation.

**Table 2:** Surgical Characteristics.

Surgical Variables		*n* (%) N = 43
Type of Surgery	Hysterectomy ± BSO	21 (48.8)
	Hysterectomy ± BSO + Pelvic ± Para-aortic LND	7 (16.3)
	Hysterectomy ± BSO ± Pelvic ± Para-aortic LND + Omentectomy	11 (25.6)
	Debulking Surgery	4 (9.3)
Surgical Approach	Laparotomy	34 (79.1)
	Laparoscopy	8 (18.6)
	Robotic Surgery	1 (2.3)
Lymphadenectomy (LND) Status	None	21 (48.8)
	Pelvic Lymphadenectomy	9 (20.9)
	Pelvic and Para-aortic Lymphadenectomy	13 (30.2)
Omentectomy Status	Not Performed	28 (65.1)
	Performed	15 (34.9)

BSO: Bilateral Salpingo-Oophorectomy, LND: lymph node dissection.

**Table 3:** Histopathological Characteristics and FIGO Staging of the Patient Cohort (N = 43).

Variables		*n* (%) or Mean ± SD
FIGO Stage (2009)	IA	16 (37.2)
	IB	13 (30.2)
	IC	5 (11.6)
	IIA	3 (70)
	IIB	1 (2.3)
	IIIA	1 (2.3)
	IIIC	4 (9.3)
Tumor Size (mm)		67.49 ± 33.04
<50 mm		13 (30.2)
≥50 mm		30 (69.8)
Myometrial Invasion	None	17 (39.5)
	<50%	13 (30.2)
	≥50%	13 (30.2)
Peritoneal Cytology	Not performed	27 (62.8)
	Negative for malignancy	16 (37.2)
Sarcomatous Component Type	Homologous	33 (76.7)
	Heterologous	10 (23.3)
Serosal Involvement		6 (14.0)
Endocervical Involvement		7 (16.3)
Sarcomatous Overgrowth		19 (44.1)
Tumor Necrosis		8 (18.6)
Lymphovascular Space Invasion		8 (18.6)
Adnexal Involvement		4 (9.3)
Lymph Node Involvement		4 (9.3)
Concomitant Gynecological Conditions	Leiomyoma	18 (41.9)
	Adenomyosis	11 (25.6)
	Endometriosis	1 (2.3)
	None	13 (30.2)

FIGO, International Federation of Gynaecology and Obstetrics, SD: Standard Deviation.

**Table 4:** Adjuvant Treatment and Recurrence Characteristics (N = 43).

Variables		*n* (%)
Adjuvant Treatment	None	19 (44.2)
	Chemotherapy alone	14 (32.6)
	Radiotherapy alone	7 (16.3)
	Chemoradiotherapy	3 (7.0)
Chemotherapy Regimen (*n* = 17)	Doxorubicin	10 (58.8)
	Gemcitabine + Docetaxel	3 (17.6)
	Carboplatin + Paclitaxel	2 (11.8)
	Ifosfamide + Mesna + Adriamycin (IMA)	1 (5.9)
	Pazopanib	1 (5.9)
Radiotherapy Modality (*n* = 10)	External beam radiotherapy	5 (50.0)
	Brachytherapy alone	1 (10.0)
	External beam radiotherapy + brachytherapy	4 (40.0)
Recurrence Rate		15 (34.9)
Recurrence Site	Pelvic	9 (60.0)
	Thoracic	3 (20.0)
	Local	1 (6.7)
	Abdominal	1 (6.7)
	Bone	1 (6.7)
Treatment at First Recurrence (*n* = 13)	Secondary cytoreductive surgery	6 (46.2)
	Chemotherapy	5 (38.5)
	Radiotherapy	2 (15.4)
Second recurrence rate		1 (4.3)

**Table 5:** Univariate Log-Rank and Multivariate Cox Regression Analyses of Prognostic Factors for Disease-Free and Overall Survival in Uterine Adenosarcoma.

Univariate Log-Rank Analyses					
Variables		Disease-Free Survival (Months) Mean ± SE	*p* ^1^	Overall Survival (Months) Mean ± SD	*p* ^2^
FIGO stage	IA–IC	132.44 ± 11.83	**<0.001**	176.54 ± 13.67	**0.003**
	≥II	25.24 ± 7.13		61.33 ± 18.76	
Sarcomatous overgrowth	Absent	142.04 ± 12.92	**0.005**	199.57 ± 10.05	**0.002**
	Present	65.24 ± 14.08		79.78 ± 14.05	
LVSI	Absent	129.04 ± 11.95	**<0.001**	176.79 ± 13.59	**0.001**
	Present	26.35 ± 8.32		53.25 ± 19.46	
Myometrial invasion	None/<50%	122.74 ± 13.33	0.139	172.38 ± 15.16	0.086
	≥50%	61.33 ± 16.56		81.23 ± 15.40	
Tumor size	<50 mm	146.77 ± 15.81	0.068	158.00 ± 12.49	0.138
	≥50 mm	94.38 ± 14.75		144.98 ± 17.74	
Tumor necrosis	Absent	121.10 ± 13.09	0.162	131.84 ± 12.06	0.869
	Present	71.13 ± 21.49		164.17 ± 28.06	
Adnexal involvement	Absent	119.80 ± 12.15	**0.026**	164.95 ± 14.10	0.142
	Present	28.33 ± 11.67		68.25 ± 28.00	
Lymph node involvement	Absent	119.38 ± 12.19	**0.028**	164.85 ± 14.12	0.154
	Present	29.75 ± 11.20		45.25 ± 15.38	
**Multivariate Cox Regression Analyses**					
**Variables**		**Disease-Free Survival HR (95% CI)**	***p***	**Overall Survival HR (95% CI)**	***p***
Sarcomatous overgrowth (Present vs. Absent)		4.41 (1.21–16.08)	**0.025**	10.23 (1.16–89.80)	**0.036**
LVSI (Present vs. Absent)		4.57 (0.77–27.10)	0.094	11.17 (1.34–93.14)	**0.026**
Adnexal involvement (Present vs. Absent)		2.11 (0.34–13.09)	0.423	0.67 (0.07–6.00)	0.719
Lymph node involvement (Present vs. Absent)		0.85 (0.11–6.71)	0.876	0.24 (0.02–2.56)	0.239

DFS, disease-free survival; FIGO: International Federation of Gyneacology and Obstetrics; HR, hazard ratio; OS, overall survival; LVSI, lymphovascular space invasion; SD, standard deviation; SE, standard error. *p* ^1^: Log-rank test for disease-free survival. *p* ^2^: Log-rank test for overall survival. Bold means statistically significant.

## Data Availability

The raw data supporting the conclusions of this article will be made available by the authors on request.

## Supplementary Materials

The following supporting information can be downloaded at https://www.mdpi.com/article/10.3390/cancers18182903/s1, Supplementary Table S1: Immunohistochemical and Proliferative Profiles of the Epithelial and Sarcomatous Components.
