# Supplementary material for: Clinical Outcomes and Prognostic Factors in Uterine Adenosarcoma: A Multicenter Retrospective Cohort Study
Source: Cancers (Basel). 2026 Sep 8;18(18):2903. doi: 10.3390/cancers18182903 (PMC13604812; doi:10.3390/cancers18182903)
Supplement: Supplementary file 1 [file cancers-18-02903-s001.zip › cancers-4532203-supplementary.pdf]

Supplementary Files

Supplementary Table S1: **Immunohistochemical and Proliferative Profiles of the Epithelial and Sarcomatous Components**

Variables		N	Mean ± SD or Median (Min–Max) or n(%)
Mitotic index (per 10 HPF)		21	7 (1–35)
Ki-67(%)	epithelial component	16	23.6 ± 17.5
	sarcomatous component	12	20 (1–80)
ER (%)	epithelial component	15	80 (0–90)
	sarcomatous component	15	40 (0–90)
PR (%)	epithelial component	12	60 (0–90)
	sarcomatous component	12	15 (0–90)
CD10		29	26 (89.7)
Cyclin D1	epithelial component	7	3 (42.9)
	sarcomatous component	11	3 (27.3)
Vimentin	epithelial component	3	2 (66.7)
	sarcomatous component	4	3 (75.0)

ER, Estrogen Receptor; HPF, High-Power Field; PR, Progesterone Receptor; SD, Standard Deviation
